# Parasite Microbiome Project: Systematic Investigation of Microbiome Dynamics within and across Parasite-Host Interactions

**DOI:** 10.1128/mSystems.00050-17

**Published:** 2017-07-18

**Authors:** Nolwenn M. Dheilly, Daniel Bolnick, Seth Bordenstein, Paul J. Brindley, Cédric Figuères, Edward C. Holmes, Joaquín Martínez Martínez, Anna J. Phillips, Robert Poulin, Karyna Rosario

**Affiliations:** a School of Marine and Atmospheric Sciences, Stony Brook University, Stony Brook, New York, USA; b Department of Integrative Biology, University of Texas at Austin, Austin, Texas, USA; c Departments of Biological Sciences and Pathology, Microbiology, and Immunology, Vanderbilt Institute for Infection, Immunology and Inflammation, Vanderbilt Genetics Institute, Vanderbilt University, Nashville, Tennessee, USA; d Department of Microbiology, Immunology, and Tropical Medicine, Research Center for Neglected Diseases of Poverty, School of Medicine & Health Sciences, The George Washington University, Washington, DC, USA; e Marie Bashir Institute for Infectious Diseases and Biosecurity, Charles Perkins Centre, School of Life and Environmental Sciences and Sydney Medical School, The University of Sydney, Sydney, NSW, Australia; f Bigelow Laboratory for Ocean Sciences, East Boothbay, Maine, USA; g Department of Invertebrate Zoology, National Museum of Natural History, Smithsonian Institution, Washington, DC, USA; h Department of Zoology, University of Otago, Dunedin, New Zealand; i College of Marine Science, University of South Florida, St. Petersburg, Florida, USA

**Keywords:** ecology, microbiome, parasitology

## Abstract

Understanding how microbiomes affect host resistance, parasite virulence, and parasite-associated diseases requires a collaborative effort between parasitologists, microbial ecologists, virologists, and immunologists. We hereby propose the Parasite Microbiome Project to bring together researchers with complementary expertise and to study the role of microbes in host-parasite interactions. Data from the Parasite Microbiome Project will help identify the mechanisms driving microbiome variation in parasites and infected hosts and how that variation is associated with the ecology and evolution of parasites and their disease outcomes. This is a call to arms to prevent fragmented research endeavors, encourage best practices in experimental approaches, and allow reliable comparative analyses across model systems. It is also an invitation to foundations and national funding agencies to propel the field of parasitology into the microbiome/metagenomic era.

## Abstract (precis)

Understanding how microbiomes affect host resistance, parasite virulence, and parasite-associated diseases requires a collaborative effort between parasitologists, microbial ecologists, virologists, and immunologists. We hereby propose the Parasite Microbiome Project to bring together researchers with complementary expertise and to study the role of microbes in host-parasite interactions.

## COMMENTARY

Characterizations of parasite diversity and interactions with hosts as well as the development of effective control methods are among the chief goals of parasitology. In an era in which microbes (archaea, bacteria, fungi, protozoans, and viruses) are known to play varied roles in host health, Koch’s postulates are notably under reconsideration in light of the effects of the microbiome and polymicrobial infections on disease (1, 2). Although researchers have historically focused on pathogenic aspects of microbes, it is now recognized that microbial communities within an organism can be beneficial and essential to an individual’s health and may even determine susceptibility or resistance to an infectious agent (3, 4). Therefore, new challenges face parasitology that can be addressed through microbial ecology approaches. This realization has propelled numerous large-scale microbiome projects, including the Human Microbiome Project and the Earth Microbiome Project, to better understand the microbiome in both healthy and disease states (NIH Human Microbiome Project Roadmap Project [http://www.ncbi.nlm.nih.gov/bioproject/43021] and The Earth Microbiome Project data site [http://www.earthmicrobiome.org/protocols-and-standards/]). These studies have led to important advances in many other disciplines, including medical and environmental science, technology, philosophy, education, and engineering.

More poorly understood, however, is the diversity, composition, and role of microbiomes within or on parasites (with the latter defined as an organism that lives, or replicates, in or on a host organism at the host’s expense). The interactions between parasites and their microbial associates may themselves impact disease outcomes and are also not well resolved. Parasitic microbes that are integrated members of the host-associated microbiome can either harbor their own associated microbiome or cause changes in the resident host-associated microbiome in a complex set of potentially nested interactions. Given the importance of understanding parasite biology and host-associated microbiomes for human health, agriculture, aquaculture, and environmental management, we propose the Parasite Microbiome Project (PMP). With an initial focus on eukaryotic parasites, the PMP aims to fill important gaps in our understanding of parasite-microbe associations and the outcomes of parasitic infection by characterizing, across space and time, (i) the microbiome (including virome) composition of parasites, and (ii) the microbiome of parasite-infected host tissues. Comparative data analyses will include the following: microbiomes within and among parasite species, the effects of different parasites on their hosts, host- and parasite-associated microbiomes, microbiomes of parasites coinfecting the same host, and intermediate host- and definitive host-associated microbiomes for parasites with complex life cycles.

Along with others, we have begun to investigate host-parasite-microbe interactions and independently confirm that parasite-microbe interactions participate in parasite ecology and disease manifestations. Parasites spanning all major groups, including bacteria, fungi, viruses, arthropods, and worms, have been documented to disrupt their host microbiome (5). However, it often remains unknown whether the disruption of the host microbiome is beneficial for the parasite, participates in the host defense mechanism against the parasite, or is merely a by-product of infection. Parasite-microbe interactions may not always be adaptive for the parasite but could nevertheless be relevant for the disease that they cause. Moreover, some parasites carry their own microbes, a parasite-associated microbiome, that in turn may influence a given infection or parasite-host interaction. Thus far, the known roles of parasite-associated microbes in host disease are diverse, ranging from enhanced nutritional environment (6), behavioral manipulation (7–9), increased inflammatory responses (10, 11), reduced host defenses (12), and carcinogenesis (13–15). Parasites can also be vectors of other pathogenic agents (16), and symbionts of parasites can interfere with the transmission of pathogens (17). Yet, parasite microbiomes remain mostly uncharacterized. As a result, the potential effects of parasites on pathogenesis and disease due to disturbance of the host’s microbiome have yet to be fully explored, and the role of parasite-associated microbes in disease development and parasite evolution has arguably been underestimated.

Therefore, the central goal of the PMP is to further propel parasitology forward by characterizing the microbiomes of parasites from undersampled representative phyla across the tree of life and elucidating their interactions with host-associated microbes and functions throughout the parasite life cycle. Through the PMP collaborative effort, researchers will identify which parasite-associated microbes have a direct or indirect role in causing disease and whether there has been a parallel change in parasite-associated microbes or microbiomes with the evolution of parasites and hosts. The project will also shed light on the role of parasites as vectors of microbes among intermediate and definitive hosts, on the dynamics of horizontal transmission of microbes between hosts and parasites, and on the corresponding impacts on parasite transmission and disease.

As an initial approach, we will launch a large-scale sequencing campaign (including targeted surveys and metagenomes) that will use standardized methodologies (e.g., sample handling and metadata collection, DNA and RNA extraction, and sequencing approaches) in line with the Unified Microbiome Initiative (18), solicit donations of samples from researchers around the world, and collaborate with existing open-source analytical platforms with cost-free open and unrestricted access to ensure that the data are available immediately upon completion of the analysis. We will also solicit collaboration with the Genomic Standard Consortium (19) and other initiatives to conduct large-scale comparative genomic studies. In addition, we will develop a partnership with natural history collections and live culture collections. For instance, nucleic acid samples and corresponding molecular voucher specimens will be preserved and curated in a permanent, scientific collection, ensuring the availability of the samples to the scientific community for reanalysis in the future. When feasible, culture isolates of parasites and their microbes, together and independently, will be maintained to allow complementary functional investigation of the mechanisms and consequences of the association on diseases or the host. Finally, we will take advantage of the growing number of available parasite genomes and transcriptomes to computationally extract information on the presence of viruses associated with parasitic eukaryotes, viruses that may be substantial, diverse, and with a long evolutionary history (20). As of today, more than 200 genomes and 150 transcriptomes of at least 200 eukaryotic parasites have been sequenced and stored in data repositories like the Sequence Read Archive (SRA) (https://www.ncbi.nlm.nih.gov/sra/).

This large-scale collaborative project will enable translation of this new paradigm to the fields of parasitology, immunology, epidemiology, resource management, and applied medicine. This effort will be achieved through the coordinated collaboration of parasitologists, microbial ecologists, virologists, immunologists, and computational biologists. The PMP will trigger and support functional approaches in parasite systems of interest, thereby leading to opportunities for using parasite-associated microbes as an indicator of system health and novel therapeutics. At a time when we are increasingly exploring the potential of probiotic supplementation, the PMP will provide a baseline for host and parasite microbiomes that will allow us to explore the beneficial and detrimental effects of these probiotic microbes on parasites and hosts. Given that there are more parasitic species on Earth than free-living organisms (21), the PMP will contribute significant data toward characterizing the biodiversity of our planet.
